# The associations of DNA methylation alterations in oxidative stress-related genes with cancer incidence and mortality outcomes: a population-based cohort study

**DOI:** 10.1186/s13148-018-0604-y

**Published:** 2019-01-24

**Authors:** Xīn Gào, Yan Zhang, Barbara Burwinkel, Yang Xuan, Bernd Holleczek, Hermann Brenner, Ben Schöttker

**Affiliations:** 1 Division of Clinical Epidemiology and Ageing Research, German Cancer Research Center, Im Neuenheimer Feld 581, 69120 Heidelberg, Germany; 2 Network Aging Research, University of Heidelberg, Bergheimer Straße 20, 69115 Heidelberg, Germany; 3 Division of Molecular Epidemiology, German Cancer Research Center, Im Neuenheimer Feld 581, 69120 Heidelberg, Germany; 4 Division Molecular Biology of Breast Cancer, University Women’s Clinic, Heidelberg University, Voßstraße 9, 69115 Heidelberg, Germany; 5 Saarland Cancer Registry, Krebsregister Saarland, Präsident-Baltz-Straße 5, 66119 Saarbrücken, Germany; 6 Division of Preventive Oncology, German Cancer Research Center (DKFZ) and National Center for Tumor Diseases (NCT), Im Neuenheimer Feld 410, 69120 Heidelberg, Germany; 7 German Cancer Consortium (DKTK), German Cancer Research Center (DKFZ), Im Neuenheimer Feld 410, 69120 Heidelberg, Germany

**Keywords:** Oxidative stress, DNA methylation, Neoplasm, Mortality, *ALOXE3*, *MTOR*, 8-isoprostane

## Abstract

**Background:**

Reactive oxygen species may be involved in epigenetic gene activation or silencing. We aimed to identify CpG sites, at which DNA methylation is related to urinary 8-isoprostane levels (biomarker of lipid peroxidation) and cancer or mortality outcomes. This investigation was based on a German, population-based cohort with linkage to cancer and mortality registry data (2000–2016).

**Results:**

Blood DNA methylation in promoter regions of 519 genes, known to be involved in pathways from oxidative stress (OS) to cancer, was obtained at the cohort's baseline examination. Inverse associations of DNA methylation at cg25365794 (*ALOXE3*) and cg08862778 (*MTOR*) with 8-isoprostane levels were observed in a derivation set (*n* = 1000) and validated in two independent subsets of the cohort (*n* = 548 and *n* = 741). Multivariate regression models were used to evaluate the associations of DNA methylation at the two CpG sites with lung, colorectal, prostate, breast, and overall cancer incidence as well as CVD, cancer, and all-cause mortality. DNA methylation at cg25365794 (*ALOXE3*) was inversely associated with lung and prostate cancer incidence. DNA methylation at cg08862778 (*MTOR*) was associated with a 43% lower breast cancer incidence in the top vs. bottom tertile.

**Conclusion:**

The finding for *ALOXE3* may not be causal. As *ALOXE3* is mainly expressed in skin tissue, the observed association might reflect the fact that both DNA methylation at the *ALOXE3* gene and urinary 8-isoprostane concentrations depend on the level of OS in tissues. Contrarily, the finding for the MTOR gene and breast cancer is biologically plausible because the MTOR protein plays an important role in PI3K/Akt signaling, which is a pathway related to cancer development and cell senescence.

**Electronic supplementary material:**

The online version of this article (10.1186/s13148-018-0604-y) contains supplementary material, which is available to authorized users.

## Background

Oxidative stress (OS) refers to an imbalance between antioxidants and oxidants production in favor of the latter, resulting in an interruption of redox signaling and control and/or molecular damage [1]. OS at physiological levels is termed oxidative eustress, and maintenance of oxidative eustress is crucial for redox regulation [2, 3]. Excessive OS, however, can damage proteins, lipids, and nucleic acids, can contribute to the development of various age-related diseases, including cancer [4]. The major oxidants are reactive oxygen species (ROS), which cannot be detected directly in human specimens because of their short half-lives [5]. Instead, elevated ROS can be indirectly measured by using oxidatively generated metabolites of proteins, lipids, or nucleic acids. The concentration of 8-isoprostane molecules in urine, a biomarker of lipid peroxidation, is the acknowledged gold standard for measurement of the oxidative stress burden of the human organism [6, 7]. Elevated 8-isoprostane levels have been shown to be a risk factor for lung cancer [8, 9].

Excessive ROS has been shown to be involved in the changes of DNA methylation levels [10, 11]. For instance, hydrogen peroxide acts as a nucleophile to deprotonate the cytosine molecule at the C-5 position, which accelerates the reaction of DNA with the positive-charged intermediate S-adenosyl-L-methionine in the process of DNA methylation [12]. In addition, ROS may regulate the expression of DNA methyltransferases (DNMT), enzymes catalyzing the transfer of a methyl group to DNA [13]. Conversely, DNA methylation alterations may regulate the expression of OS-related genes [14]. In spite of biological plausibility, evidence from human population-based studies on the associations of OS biomarker concentrations with DNA methylation of OS-related genes is sparse and the potential impact of OS-related DNA methylation alteration on cancer development is even sparser [15, 16].

Therefore, this study aimed (i) to conduct a gene-specific screening for CpG sites, differentially methylated according to urinary 8-isoprostane levels, and (ii) to investigate whether identified CpG sites are associated with the risk of cancer or mortality.

## Methods

### Study population

This study is based on the ESTHER study (German: *Epidemiologische Studie zu Chancen der Verhütung, Früherkennung und optimierten Therapie chronischer Erkrankungen in der älteren Bevölkerung*), which is an ongoing prospective, population-based cohort study. Details of the study design have been reported elsewhere [17, 18]. Briefly, general practitioners, (GPs) recruited 9949 study participants aged 50 to 74 years, during a general health check-up between 2000 and 2002 in Saarland, a federal state of Germany. Spot urine and whole blood samples were collected during the health check-up, shipped to the study center, and maintained at − 80 °C until further processing.

**Fig. 1:**
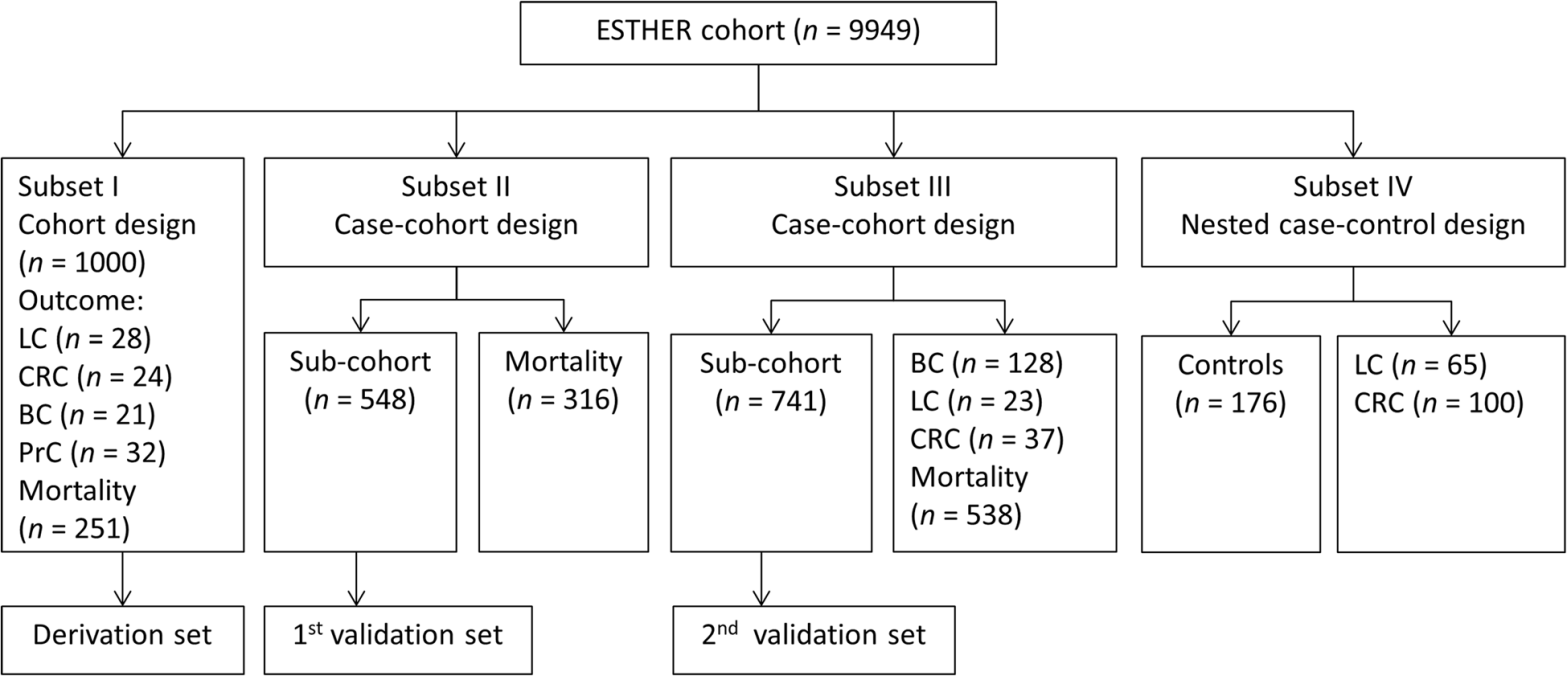
Four subsets of the German ESTHER study selected for measurement of epigenome-wide DNA methylation data, Abbreviation: BC, breast cancer; CRC, colorectal cancer; LC, lung cancer

Four independent subsets were selected from the ESTHER study for epigenome-wide DNA methylation data measurements for various projects [19] and were used for the current analysis (Fig. 1). Subset I includes the first 500 recruited men and the first 500 recruited women (recruited between July and October 2000). Subset II has a case-cohort design for mortality with *n* = 316 deaths and a random sub-cohort of *n* = 548. Subset III has a case-cohort design with *n* = 128 breast cancer, *n* = 58 lung cancer, *n* = 23 colorectal cancer, and *n* = 538 mortality cases and a random sub-cohort with *n* = 741 subjects. Subset IV has a nested case-control design with *n* = 65 incident lung cancer cases, *n* = 100 colorectal cancer cases, and *n* = 176 controls. In the gene-specific screening for CpG sites, differentially methylated according to urinary 8-isoprostane levels, subset I was used as the derivation set and the random sub-cohorts of subset II and III were used as two independent validation sets. Finally, all four subsets were used to determine potential associations of identified CpG sites with cancer (lung, colorectal, breast, prostate, and overall cancer) and mortality outcomes (cancer, CVD, and all-cause mortality).

### Laboratory analyses

Urinary 8-isoprostane levels were determined by 8iso1 ELISA kit from Detroit R&D (Detroit, Michigan, USA) with two- or fourfold dilution, depending on the concentrations of the marker. According to the manufacturer’s manual, the specificity of the assay is 100%. For renal function adjustment of spot urine samples, urinary creatinine was determined by the kinetic Jaffe method and 8-isoprostane levels were expressed with the unit “nmol/mmol creatinine.”

DNA methylation profiles of subset I, II, and IV were assessed with the Infinium HumanMethylation450 BeadChip (450 k) array, and DNA methylation profiles of subset III were assessed with the Infinium Methylation EPIC BeadChip kit that covers 850,000 CpG sites (850k) (Illumina, San Diego, CA, USA). The assays were conducted by following the manufacturer’s instruction at the Genomics and Proteomics Core Facility of the German Cancer Research Center, Heidelberg, Germany [20]. The methylation status of a specific CpG site was quantified as a *β*-value ranging from 0 (no methylation) to 1 (full methylation). No background correction was done, and data were normalized to internal controls provided by the manufacturer. All controls were checked for inconsistencies in each measured plate. Signals of probes with a detection *P* value > 0.05 were excluded from analysis.

### Outcome ascertainment

Incident cancers until the end of 2014 were ascertained by linkage with the Saarland Cancer Registry. According to the 10th Revision of the International Statistical Classification of Diseases (ICD-10), cancer cases during follow-up were defined by all ICD-10 C-codes but C44 (non-melanoma skin cancer). Colorectal, lung, breast, and prostate cancer were defined by the ICD-10 codes C18-C21, C34, C50, and C61, respectively.

Deaths during follow-up by the end of 2015 were ascertained by inquiry at the residents’ registration offices, and information on the vital status of 99.9% of the cohort's participants could be obtained. Additionally, death certificates were provided by local health authorities for 97.7% of those who had died. All deaths coded with ICD-10 codes I00–I99 were considered cardiovascular deaths, and cancer deaths were defined by ICD-10 codes C00–C99 and D37–D48.

### Covariates assessment

Information on sociodemographic characteristics; smoking behavior; physical activity; the consumption of alcohol, fruits, vegetables, and meat; an asthma diagnosis; and history of cardiovascular events (stroke, myocardial infarction, pulmonary embolism, bypass operation, or dilatation of the coronary vessels) were obtained from a standardized self-administered questionnaire. Height, weight, and a history of diabetes or coronary heart disease (CHD) were assessed and documented on a standardized form by GPs during the health check-up. The history of cancer before baseline was determined by either self-report or record linkage with data from the Saarland Cancer Registry, which started to record cancers in 1970.

### Selection of CpG site candidates

**Fig. 2:**
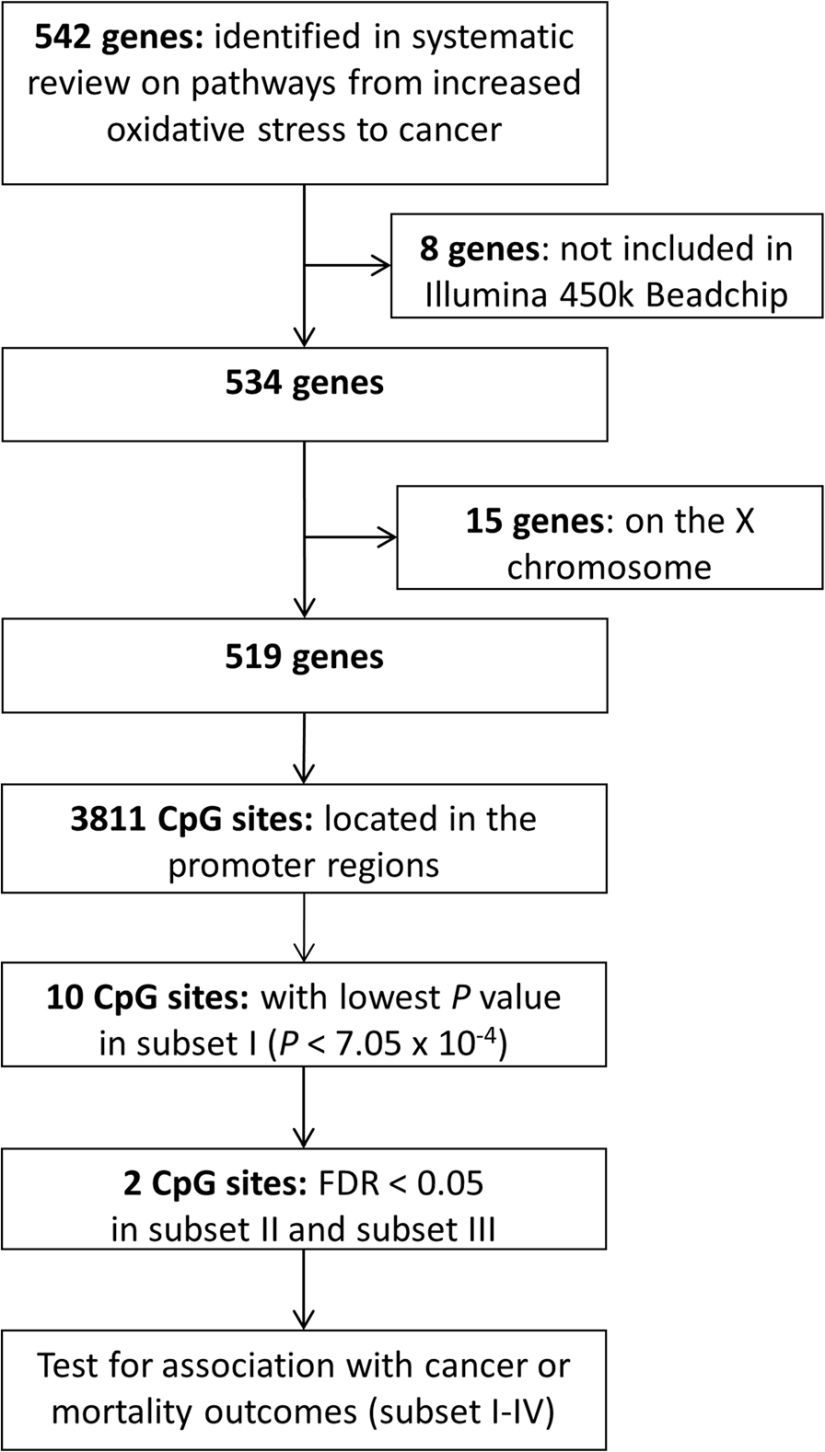
Flow chart of CpG sites selection, Abbreviation: FDR, false discovery rate

To increase the statistical power, a gene-specific search was performed with restriction to genes coding for proteins that are involved in intracellular ROS generating organelles and enzymes and signal transduction cascades kinases/phosphatases or transcription factors that are on pathways from increased OS to cancer development. These proteins have been identified by our group in a systematic literature review and reported previously [21]. Altogether, 542 genes involved in 18 pathways were identified. We excluded 8 genes, which were not included in the 450k array, and 15 genes, which were on the X chromosome, leaving 519 genes for analyses. The 3811 CpG sites in the promoter regions of these 519 genes were selected for the screening in the derivation set. The selection process of the CpG sites is illustrated in Fig. 2, and the 519 selected genes and their pathways are listed in Additional file 1: Table S1.

### Statistical analyses

Baseline characteristics of participants of the derivation sample and the two validation samples were expressed as medians (interquartile ranges) or proportions, and differences among the three samples were determined by Wilcoxon-Mann-Whitney tests for continuous variables and by chi-square tests for discrete variables.

In the derivation set, a mixed linear regression model was used to assess the associations between the methylation levels of the selected 3811 CpG sites and 8-isoprostane levels. A natural logarithm transformation of 8-isoprostane levels was employed to ensure normal distribution. The model was adjusted for age (continuously), sex (male/female), leukocyte composition using Houseman’s algorithm [22] (6 continuous variables), alcohol consumption (continuously), body mass index (BMI, continuously), physical activity (inactive, low, medium, or high), fruit consumption (</≥ once/day), vegetables consumption (</≥ once/day), meat consumption (</≥ once/day), smoking status (with seven categories, as shown in Table 1), history of cancer (yes/no), cardiovascular diseases (yes/no), diabetes (yes/no), and asthma (yes/no). Batch-specific variations of the DNA methylation assay were modeled as random effects.

**Table 1:** Baseline characteristics of study participants used for the gene-specific screening for CpG sites, differentially methylated according to urinary 8-isoprostane levels

Characteristics	Derivation set (*n* = 1000)	1st validation set (*n* = 548)	2nd validation set (*n* = 741)	*P* value^*#*^
Age (year, median, IQR)	62 (57–67)	62 (57–67)	62 (56–67)	0.30
Sex (*n*, %)				< 0.001
Female	500 (50.0)	324 (61.0)	415 (56.0)	
Male	500 (50.0)	224 (39.0)	326 (44.0)	
Education (*n*, %)				0.95
< 9 years	737 (73.7)	411 (75.0)	548 (74.0)	
9–11 years	155 (15.5)	85 (15.5)	115 (15.5)	
> 12 years	108 (10.8)	52 (9.5)	78 (10.5)	
Smoking status (*n*, %)				0.19
Never smoker	481 (48.1)	257 (46.9)	363 (49.0)	
Former smoker who quitted > 20 years ago	124 (12.4)	74 (13.5)	112 (15.1)	
Former smoker who quitted 5 to < 20 years ago	149 (14.9)	83 (15.2)	100 (13.5)	
Former smoker who quitted 0 to < 5 years ago	60 (6.0)	29 (5.3)	31 (4.2)	
Current smoker smoking 0 to < 15 g tobacco/day	84 (8.4)	49 (8.9)	60 (8.1)	
Current smoker smoking 15 to < 30 g tobacco/day	97 (9.7)	49 (8.9)	60 (8.1)	
Current smoker smoking > 30 g tobacco/day	5 (0.5)	7 (1.3)	15 (2.0)	
Alcohol consumption (g/day, median, IQR) ^†^	6.0 (0–14.4)	5.6 (0–12.6)	4.8 (0–13.7)	0.80
Physical activity (*n*, %) ^‡^				0.17
Inactive	204 (20.4)	114 (20.8)	157 (21.2)	
Sedentary	438 (43.8)	270 (49.3)	343 (46.3)	
Vigorously active	358 (35.8)	164 (39.9)	241 (32.5)	
BMI (kg/m^2^, median, IQR)	27.4 (25.0–30.1)	27.1 (24.5–30.1)	27.2 (24.9–30.1)	0.39
Categorized BMI (*n*, %)				< 0.001
< 25 kg/m^2^	251 (25.1)	167 (30.5)	195 (26.3)	
25 to < 30 kg/m^2^	483 (48.3)	235 (42.9)	350 (47.2)	
≥ 30 kg/m^2^	266 (26.6)	146 (26.6)	196 (26.5)	
Dietary factors (*n*, %)				
Fruit consumption, < once/day	391 (39.2)	192 (35.0)	287 (38.7)	0.26
Vegetable consumption, < once/day	656 (65.6)	341 (62.2)	461 (62.2)	0.25
Meat consumption, ≥ once/day	349 (34.9)	178 (31.5)	247 (33.3)	0.59
Leukocyte composition (mean, SD) ^┼^				
CD8+ T cells	0.07 (0.05–0.10)	0.09 (0.07–0.12)	0.06 (0.04–0.09)	< 0.001
CD4+ T cells	0.16 (0.12–0.20)	0.17 (0.13–0.20)	0.16 (0.12–0.20)	0.01
NK cells	0.91 (0.07–0.12)	0.09 (0.07–0.12)	0.10 (0.07–0.13)	0.18
B cells	0.06 (0.05–0.07)	0.07 (0.06–0.08)	0.06 (0.04–0.07)	< 0.001
Monocytes	0.10 (0.09–0.11)	0.10 (0.09–0.11)	0.07 (0.06–0.08)	< 0.001
Granulocytes	0.55 (0.48–0.60)	0.54 (0.47–0.59)	0.56 (0.50–0.62)	< 0.001
8-isoprostane (nmol/mmol creatinine, median, IQR)	0.19 (0.13–0.26)	0.21 (0.16–0.27)	0.21 (0.16–0.28)	< 0.001
Prevalent cancer cases (*n*, %)	75 (7.5)	32 (6.0)	57 (7.7)	0.46
Prevalent CVD cases (*n*, %) ^^^	218 (21.8)	107 (20.2)	137 (18.5)	0.24
Prevalent diabetes cases (*n*, %)	155 (15.5)	89 (16.8)	108 (14.6)	0.64
Prevalent asthma cases (*n*, %)	74 (7.4)	23 (4.3)	49 (6.6)	0.19

*Abbreviations*: *BMI* body mass index, *CVD* cardiovascular disease, *IQR* interquartile range

^**#**^Differences in baseline characteristics among the three groups were assessed with a Wilcoxon signed-rank test for continuous variables and a chi-square test for categorical variables

^†^The consumption of alcohol was calculated by the following equation: 1 bottle of beer = 11.88 g ethanol, 1 glass of wine = 22.0 g ethanol, 1 shot of liquor = 6.4 g ethanol

^‡^Definition of inactive: < 1 h of physical activity/week. Definition of medium or high physical activity: ≥ 2 h of vigorous and ≥ 2 h of light physical activity/week. Definition of low physical activity: all other amounts of activity not categorized as “inactive” or “medium or high”

^┼^Estimated by the Houseman algorithm

^^^Cardiovascular disease at baseline is a composite variable of either coronary heart disease or history of one or more cardiovascular event (i.e., stroke, myocardial infarction, pulmonary embolism, bypass operation, or dilatation of the coronary vessels)

The top 10 CpG sites with the lowest *P* values for the association with 8-isoprostane levels in the derivation set were selected, and the same linear mixed regression model analyses were repeated in the two validation sets. Obtained *β*-coefficients and standard errors were subsequently pooled by fixed effects meta-analysis. To account for multiple testing, only CpG sites with a false discovery rate (FDR) < 0.05 were considered to be statistically significant and selected for analyses with cancer and mortality outcomes.

To explore the associations between DNA methylation at the selected CpG sites and cancer incidences (overall, lung, colorectal, breast, and prostate cancer) as well as mortality outcomes (all-cause, cancer-specific and cardiovascular disease-specific mortality), Cox regression, weighted Cox regression [20], and logistic regression models were used for cohort (subset I and random sub-cohorts of subset II and II), case-cohort (subset II and III), and nested case-control study designs (subset IV), respectively. Hazard ratios (HRs) with corresponding 95% confidence intervals (95% CIs) were estimated in subset I–III, and odds ratios (ORs) with 95% CIs were assessed in the subset IV and results were pooled by fixed effects meta-analysis. Subjects with a history of the specific cancer of interest were excluded. In a sensitivity analysis, also cancer cases that occurred in the first 2 years of follow-up (potentially undiagnosed cancers at baseline) were excluded, but overall, the results did not change (data not shown). The regression models were adjusted for age, sex, leukocyte composition, and batches. Additional potential confounders, to be adjusted for in the regression models, needed to be statistically significantly associated with DNA methylation levels at the selected CpG site (*P* < 0.05). The potential confounders were identified among all variables shown in Table 1 in general linear models. Methylation intensities were included into the models either as tertiles to test for non-linear associations (bottom tertiles defined as the reference category) or as continuous variables to test for linear associations.

Multiple imputation was applied to adequately deal with missing values, and five data sets were imputed. The variables for the imputation model were those shown in Table 1. No variable had more than 10% missing information. The multiple imputation assumption (values missing at random) was examined, and individuals with complete data did not differ from those with incomplete data (data not shown). All statistical tests were conducted with the Statistical Analysis System (SAS, version 9.4, Cary, NC, USA).

## Results

Table 1 presents the baseline characteristics of participants in derivation set and the two validation sets. Subjects were on average 62 years old. Because of different sampling strategies, the derivation set includes equal numbers of men and women and the validation sets have a higher proportion of women than men. The different sex distribution resulted in some statistically significant differences in baseline characteristics (categorized BMI, asthma, 8-isoprostane levels, leukocyte composition), but otherwise, the characteristics of the three samples were comparable.

From the 3811 CpG sites in the promoter regions of the 519 selected genes, information on the top 10 CpG sites associated with 8-isoprostane concentrations in the derivation set are shown in Additional file 1: Table S2 (*P* value ≤ 7.05 × 10^−4^; FDR ≤ 0.227) and the pooled results from the two validation sets are shown in Table 2. DNA methylation levels at three CpG sites were statistically significantly associated with 8-isoprostane concentrations after FDR correction: cg25365794 [*Arachidonate LipOXygEnase 3* (*ALOXE3*) gene], cg01009697 [*Neurotrophic Receptor Tyrosine Kinase 2* (*NTRK2*) gene], and cg08862778 [*Mechanistic target of rapamycin kinase* (*MTOR*) gene]. However, the methylation levels at cg01009697 were positively associated with 8-isoprostane levels in the deviation set (Additional file 1: Table S2) and inversely associated with 8-isoprostane levels in the validation sets (Table 2), which means that the results in the derivation set were not confirmed in the validation sets. Therefore, cg01009697 was excluded from further analyses. The distribution of the methylation levels of the selected two CpG sites in the three subsets is shown in Additional file 1: Figure S1. The linear inverse associations of the methylation levels at the two selected CpG sites with 8-isoprostane levels are additionally shown graphically in a scatter plot in Additional file 1: Figure S2. The explained variance of 8-isoprostane levels by DNA methylation at both CpG sites was rather low (*R*^2^ ranged from 0.0015 to 0.0148 in the three subsets). None of the baseline characteristics of the study participants was statistically significantly associated with DNA methylation at the two selected CpG sites (data not shown).

**Table 2:** Meta-analysis of the associations of DNA methylation at the top 10 CpG sites associated with 8-isoprostane concentrations in the two validation sets

Identified CpG sites	Gene name	Pathways	*ß*-coefficient	SE	*P* value	FDR
cg25365794	*ALOXE3*	Prostaglandin 2 biosynthesis and metabolism	− 4.540	1.338	0.001	0.010
cg01009697	*NTRK2*	PI3K/Akt and MAPK signaling pathways	− 2.266	0.780	0.004	0.020
cg08862778	*MTOR*	Various pathways, such as PI3K/Akt	− 3.326	1.270	0.009	0.030
cg27095527	*PPARG*	Nuclear receptors in lipid metabolism and toxicity	− 0.694	0.695	0.318	0.694
cg05784862	*KSR1*	RET signaling and MAPK signaling pathways	− 0.224	0.295	0.447	0.694
cg19623877	*MYB*	Response to elevated platelet cytosolic Ca^2+^	0.427	0.604	0.480	0.694
cg06671842	*PTPN5*	PAK and MAPK signaling pathways	− 0.307	0.440	0.486	0.694
cg19192120	*SSH3*	Regulation of actin cytoskeleton and cytoskeletal signaling pathways	− 0.094	0.321	0.769	0.898
cg02168857	*EPHA7*	EPHA forward signaling pathway	− 0.117	0.480	0.808	0.898
cg15093079	*EPHA6*	EPHA forward signaling pathway	0.025	0.659	0.969	0.969

*Abbreviations*: *Akt* protein kinase B, *ALOXE3* arachidonate lipoxygenase 3, *EPHA6* EPH receptor A6, *EPHA7* EPH receptor A7, *FDR* false discovery rate, *KSR1* Kinase suppressor of Ras 1, *MAPK* mitogen-activated protein kinase, *MTOR* mechanistic target of rapamycin kinase, *MYB* MYB proto-oncogene, transcription factor, *NTRK2* neurotrophic receptor tyrosine kinase 2, *PI3K* phosphoinositide 3-kinase, *PPARG* peroxisome proliferator activated receptor gamma, *PTPN5* protein tyrosine phosphatase, non-receptor type 5, *SE* standard error, *SSH3* slingshot protein phosphatase 3

The identified two CpG sites were carried forward to the testing for associations of OS-related DNA methylation with cancer and mortality outcomes (Table 3). Every one standard deviation (SD) increase in DNA methylation at cg25365794 (*ALOXE3* gene) resulted in a 19% decrease in incidence of lung cancer (HR (95%) 0.81 (0.66, 0.99)). Furthermore, an inverse association of cg25365794 (*ALOXE3* gene) with prostate cancer was observed (HR (95% CI) per 1 SD increase: 0.78 (0.60, 1.03)), but only the comparison of the middle and the bottom tertile was statistically significant (HR (95% CI) 0.47 (0.24, 0.92)). DNA methylation at cg08862778 (*MTOR* gene) was statistically inversely associated with breast cancer (top tertile vs. bottom tertile, HR (95% CI) 0.57 (0.33, 0.97)).

**Table 3:** Associations of oxidative stress-related DNA methylation at the selected CpG sites with cancer incidences and mortality outcomes

Outcomes	cg25365794	cg08862778
	HR (95% CI)*	HR (95% CI)*
Overall incident cancer^*#*^		
Tertile 1	Ref.	Ref.
Tertile 2	0.77 (0.59, 1.01)	0.93 (0.71, 1.21)
Tertile 3	0.90 (0.68, 1.18)	0.84 (0.62, 1.12)
Increase per 1 SD	0.98 (0.87, 1.10)	0.99 (0.87, 1.12)
Incident lung cancer^†^		
Tertile 1	Ref.	Ref.
Tertile 2	0.73 (0.46, 1.16)	0.90 (0.57, 1.41)
Tertile 3	0.78 (0.48, 1.27)	0.67 (0.40, 1.11)
Increase per 1 SD	*0.81 (0.66, 0.99)*	0.94 (0.77, 1.15)
Incident colorectal cancer^†^		
Tertile 1	Ref.	Ref.
Tertile 2	0.72 (0.43, 1.19)	1.07 (0.65, 1.76)
Tertile 3	0.83 (0.50, 1.40)	1.46 (0.88, 2.44)
Increase per 1 SD	0.98 (0.79, 1.21)	1.16 (0.96, 1.41)
Incident breast cancer^‡^ (in female participants)		
Tertile 1	Ref.	Ref.
Tertile 2	0.91 (0.55, 1.51)	0.67 (0.40, 1.11)
Tertile 3	0.91 (0.50, 1.65)	*0.57 (0.33, 0.97)*
Increase per 1 SD	0.90 (0.70, 1.16)	0.84 (0.63, 1.11)
Incident prostate cancer^*#*^(in male participants)		
Tertile 1	Ref.	Ref.
Tertile 2	*0.47 (0.24, 0.92)*	0.97 (0.49, 1.92)
Tertile 3	0.68 (0.37, 1.24)	1.11 (0.51, 2.42)
Increase per 1 SD	0.78 (0.60, 1.03)	1.15 (0.82, 1.62)
All-cause mortality^┼^		
Tertile 1	Ref.	Ref.
Tertile 2	0.90 (0.74, 1.11)	0.99 (0.80, 1.22)
Tertile 3	1.14 (0.93, 1.41)	1.17 (0.93, 1.47)
Increase per 1 SD	1.03 (0.94, 1.12)	1.08 (0.98, 1.18)
Cancer mortality^┼^		
Tertile 1	Ref.	Ref.
Tertile 2	0.91 (0.69, 1.20)	0.87 (0.66, 1.16)
Tertile 3	0.87 (0.65, 1.16)	0.97 (0.71, 1.31)
Increase per 1 SD	0.92 (0.81, 1.05)	1.06 (0.94, 1.20)
CVD mortality^┼^		
Tertile 1	Ref.	Ref.
Tertile 2	0.87 (0.65, 1.16)	1.04 (0.77, 1.40)
Tertile 3	1.23 (0.92, 1.66)	1.28 (0.93, 1.75)
Increase per 1 SD	1.04 (0.92, 1.17)	1.08 (0.95, 1.22)

Numbers printed in italics: statistically significantly different from 1 (*P* < 0.05)

*Abbreviations*: *CI* confidence interval, *CVD* cardiovascular disease, *HR* hazard ratio, *SD* standard deviation

*The Cox regression model was adjusted for age, sex, batch, and leukocyte distribution

^#^Meta-analyzed results from subset I (cohort design, *n* = 1000), sub-cohort of subset II (cohort design, *n* = 548), and sub-cohort of subset III (cohort design, *n* = 741)

^†^Meta-analyzed results from subset I (cohort design, *n* = 1000), sub-cohort of subset II (cohort design, *n* = 548), subset III (case-cohort design, *n* = 741; sub-cohort, *n* = 80 lung cancer cases, *n* = 37 colorectal cancer cases), and subset IV (nested case-control design, *n* = 65 lung cancer cases, *n* = 100 colorectal cancer cases, *n* = 176 controls)

^‡^Meta-analyzed results from subset I (cohort design, *n* = 1000), sub-cohort of subset II (cohort design, *n* = 548), and subset III (case-cohort design, *n* = 741; sub-cohort, *n* = 128 breast cancer cases)

^┼^Meta-analyzed results from subset I (cohort design, *n* = 1000), subset II (case-cohort design, *n* = 548; sub-cohort, *n* = 316 all-cause mortality, *n* = 128 cancer mortality, *n* = 104 CVD mortality), and subset III (case-cohort design, *n* = 741; sub-cohort, *n* = 538 all-cause mortality, *n* = 209 cancer mortality, *n* = 181 CVD mortality)

## Discussion

In summary, based on this gene-promoter-specific screening analysis in three independent subsets of the ESTHER cohort, DNA methylation at cg25365794 (*ALOXE3* gene) and cg08862778 (*MTOR* gene) where inversely associated with 8-isoprostane levels. In further analysis, association of DNA methylation at the two selected CpG sites with cancer or mortality outcomes was explored in four subsets has been meta-analyzed. DNA methylation at cg25365794 (*ALOXE3* gene) was inversely associated with lung and prostate cancer. Moreover, an inverse association was found between DNA methylation at cg08862778 (*MTOR* gene) and breast cancer.

The *ALOXE3* gene encodes arachidonate lipoxygenase 3, which converts polyunsaturated fatty acid hydroperoxides via an alkoxyl radical intermediate to epoxyalcohols and ketons [23]. These products play an indispensable role in formation of the water-impermeable barrier of the outer epidermis [24]. As *ALOXE3* is mainly expressed in skin tissue and to a lesser extent in several other tissues [25], a direct link to 8-isoprostane concentrations in urine is rather unlikely. The observed significant correlation between DNA methylation at the *ALOXE3* gene and 8-isoprostane concentrations might reflect the fact that both depend on the level of OS in tissues. Taken together, the observed associations of DNA methylation at the *ALOXE3* gene and lung and prostate cancer development might not be causal. However, the currently missing biological plausibility may be found by future studies. To our knowledge, only one population-based study linked *ALOXE3* gene and cancer so far and showed that men with mutations in the *ALOXE3* gene can have a dysfunctional epidermis barrier and may have an increased risk for prostate cancer if exposed to pesticides [26].

The *MTO*R gene encodes a serine-threonine protein kinase (mTOR) serving as a core component of two multi-protein complexes, mTOR complex 1 (mTORC1) and mTOR complex 2 (mTORC2), which respond to stressors, including DNA damage, nutrients, and oxidative stress [27, 28]. Hydrogen peroxide, a major product of oxidative stress, can activate the PI3K/AKT/mTOR signaling pathway by inhibiting its suppressor, PTEN (phosphatase and tensin homolog) [21]. The PI3K/Akt axis is involved in the regulation of the mTORC1. Activation of PI3K leads to phosphorylation and activation of Akt, which subsequently activates mTORC1 [29]. Over-activation of mTORC1 leads to widespread but benign tumor formation [28]. The mTORC2 protein acts as a tyrosine kinase and phosphorylates Akt, which has a function in maintaining cancer cell survival [30, 31]. Therefore, the PI3K/Akt/mTOR signaling is a target for the treatment of cancer. For instance, everolimus is an mTOR inhibitor and was approved for breast cancer treatment [32]. Supporting our findings, Tang et al. observed that a CpG site located in the *regulatory associated protein of MTOR complex 1* (*RPTOR*) gene, which is an activator of mTOR, was hypomethylated in breast cancer patients compared to healthy controls [33]. Therefore, our finding adds evidence to a pathway of ROS-mediated PI3K/AKT/mTOR activation to breast cancer development. This pathway might be in part controlled by DNA demethylation in the promoter region of the *MTOR* gene.

To our knowledge, our study is the first gene-specific study, which screened for associations of DNA methylation with 8-isoprostane levels. There is only one other similar study, which used a different biomarker of OS (derivatives of reactive oxygen metabolites (d-ROM)) and had a much smaller sample size (*n* = 99 in derivation set, *n* = 142 in validation set) [15]. In this epigenome-wide screening study, DNA methylation at cg10342304 (*nucleoredoxin* (*NXN*) gene) was associated with derivatives of reactive oxygen metabolites (d-ROM) and, moreover, significantly associated with overall cancer incidence [15]. A further study that measured DNA methylation in four oxidative stress-related genes identified a significant association of DNA methylation at one site in the promotor region of the *8-oxoguanine DNA glycosylase* (*OGG1*) gene with overall cancer incidence and prostate cancer incidence [16]. However, the results were neither corrected for multiple testing nor validated in another sample. The choice of different OS biomarkers in these two studies may explain why we did not observe associations with DNA methylation at the *NXN* and the *OGG1* gene.

Our analysis has a number of strengths. First, detailed information on a broad range of covariates enabled us to control for potential confounding as far as possible. Second, its prospective design for the cancer outcomes precluded reverse causality. Third, usage of almost complete cancer registry and mortality follow-up data excluded misclassification and non-response bias. Fourth, we performed the screening with OS and cancer-related genes identified in a systematic review. This hypothesis-based gene-specific approach has a higher statistical power than an epigenome-wide screening approach. Lastly, the 8-isoprostane molecule has proven long-term stability in frozen urine samples and is a reliable biomarker for OS [34]. Nevertheless, DNA methylation at cg25365794 (*ALOXE3* gene) and cg08862778 (*MTOR* gene) may be useful in the future as biomarkers for long-term OS exposure because they may have a higher intra-individual stability time than urinary concentrations of 8-isoprostane.

However, several limitations of our analysis should be taken into account when interpreting the results. First, DNA methylation levels vary across tissue types [35] and whole blood DNA methylation can only reflect the overall methylation levels in leukocytes. Second, screening and validation sets were obtained from the same study population. Other studies are needed to corroborate our findings for the *ALOXE3* and *MTOR* genes, to conduct gene expression analyses in multiple CpG sites in the promotor/CpG islands of these genes, and to find further CpG sites with OS-related DNA methylation. This may provide further insights into the mechanisms of OS-related cancer development and aging.

## Conclusion

In this population-based cohort, DNA methylation at two CpG sites was associated with urinary 8-isoprostane levels. DNA methylation alterations at the identified CpG sites were associated with specific cancer outcomes. While the association between urinary 8-isoprostane levels and DNA methylation at the *ALOXE3* gene may not be causal, the findings for *MTOR* gene methylation are biologically plausible. There might be a pathway of ROS-mediated PI3K/AKT/mTOR activation to breast cancer that is in part controlled by DNA demethylation in the promoter region of the *MTOR* gene.

## Additional file

Additional file 1: **Table S1.** 519 genes coding for proteins involved in pathways from oxidative stress to cancer. **Table S2.** Top 10 CpG sites with respect to associations with 8-isoprostane concentrations in the derivation set. **Figure S1.** Distributions of the methylation levels of the selected CpG sites across subsets. **Figure S2.** Scatter plots showing linear associations between DNA methylation at two selected CpG sites and 8-isoprostane levels (log-transformation) across subsets. (DOCX 744 kb)
