# Supplementary material for: The associations of DNA methylation alterations in oxidative stress-related genes with cancer incidence and mortality outcomes: a population-based cohort study
Source: Clin Epigenetics. 2019 Jan 24;11:14. doi: 10.1186/s13148-018-0604-y (PMC6346508; doi:10.1186/s13148-018-0604-y)
Supplement: Supplementary file 1 — Table S1. 519 genes coding for proteins involved in pathways from oxidative stress to cancer. Table S2. Top 10 CpG sites with respect to associations with 8-isoprostane concentrations in the derivation set. Figure S1. Distributions of the methylation levels of the selected CpG sites across subsets. Figure S2. Scatter plots showing linear associations between DNA methylation at two selected CpG sites and 8-isoprostane levels (log-transformation) across subsets. (DOCX 744 kb) [file 13148_2018_604_MOESM1_ESM.docx]

**Supplementary Material**

**Table S1 519 genes coding for proteins involved in pathways from oxidative stress to cancer**

| **Gene name** | **Pathway** |
| --- | --- |
| *AATK* | PTK |
| *ABL1* | PTK |
| *ABL2* | PTK |
| *ACP1* | PTP |
| *AHR* | TFs |
| *AKT1* | PI3K |
| *AKT2* | PI3K |
| *AKT3* | PI3K |
| *ALK* | PTK |
| *ALOX12* | ROS Source |
| *ALOX12B* | ROS Source |
| *ALOX15* | ROS Source |
| *ALOX15B* | ROS Source |
| *ALOX5* | ROS Source |
| *ALOXE3* | ROS Source |
| *AOX1* | Superpathway of tryptophan utilization |
| *APC* | Wnt/Beta-catenin |
| *APEX1* | APE/Ref-1 |
| *ARNT* | HIF |
| *ARNT2* | HIF |
| *ARNTL* | HIF |
| *ATF1* | AP-1 |
| *ATF2* | AP-1 |
| *ATF3* | AP-1 |
| *ATF4* | AP-1 |
| *ATF5* | AP-1 |
| *ATF6* | AP-1 |
| *ATF6B* | AP-1 |
| *ATF7* | AP-1 |
| *ATM* | PCK |
| *AXIN1* | Wnt/Beta-catenin |
| *AXL* | PTK |
| *BACH1* | Nrf2 |
| *BAD* | NfκB |
| *BATF* | AP-1 |
| *BAX* | NfκB |
| *BCL2* | NfκB |
| *BCL2A1* | NfκB |
| *BLK* | PTK |
| *BRAF* | MAPK |
| *BTRC* | Nrf2 |
| *CA1* | Nrf2 |
| *CA12* | Nrf2 |
| *CA13* | Nrf2 |
| *CA14* | Nrf2 |
| *CA2* | Nrf2 |
| *CA3* | Nrf2 |
| *CA4* | Nrf2 |
| *CA5A* | Nrf2 |
| *CA6* | Nrf2 |
| *CA7* | Nrf2 |
| *CA9* | Nrf2 |
| *CAT* | Antioxidant enzymes |
| *CCND2* | NfκB |
| *CCND3* | NfκB |
| *CD209* | NfκB |
| *CDC14A* | PTP |
| *CDC14B* | PTP |
| *CDC25A* | PTP |
| *CDC25B* | PTP |
| *CDC25C* | PTP |
| *CDC37* | PI3K |
| *CDH1* | Nrf2 |
| *CDKN1A* | Nrf2 |
| *CDKN3* | PTP |
| *CES1* | Nrf2 |
| *CES2* | Nrf2 |
| *CES3* | Nrf2 |
| *CES4* | Nrf2 |
| *CES7* | Nrf2 |
| *CES8* | Nrf2 |
| *CFLAR* | NfκB |
| *CHUK* | NfκB |
| *CITED2* | HIF |
| *CRAT* | Peroxisomal lipid metabolism |
| *CREB1* | APE_Ref_1 |
| *CREB3* | APE_Ref_1 |
| *CREB3L1* | APE_Ref_1 |
| *CREB3L2* | APE_Ref_1 |
| *CREB3L3* | APE_Ref_1 |
| *CREB3L4* | APE_Ref_1 |
| *CREB5* | APE_Ref_1 |
| *CREBBP* | Nrf2 |
| *CSF1R* | PTK |
| *CSK* | PTK |
| *CTNNB1* | Wnt Beta catenin |
| *CUL3* | Nrf2 |
| *DDR1* | PTK |
| *DDR2* | PTK |
| *DNAJC12* | AP_1 |
| *DPP3* | Nrf2 |
| *DUOX1* | ROS Source |
| *DUOX2* | ROS Source |
| *DUSP1* | PTP |
| *DUSP10* | PTP |
| *DUSP11* | PTP |
| *DUSP12* | PTP |
| *DUSP13* | PTP |
| *DUSP14* | PTP |
| *DUSP15* | PTP |
| *DUSP16* | PTP |
| *DUSP18* | PTP |
| *DUSP19* | PTP |
| *DUSP2* | PTP |
| *DUSP22* | PTP |
| *DUSP23* | PTP |
| *DUSP26* | PTP |
| *DUSP27* | PTP |
| *DUSP3* | PTP |
| *DUSP4* | PTP |
| *DUSP5* | PTP |
| *DUSP6* | PTP |
| *DUSP7* | PTP |
| *DUSP8* | PTP |
| *EGFR* | PTK |
| *EGLN1* | HIF |
| *EGLN2* | HIF |
| *EGLN3* | HIF |
| *EGR1* | APE/Ref-1 |
| *EIF2AK3* | Nrf2 |
| *EIF4EBP1* | HIF |
| *ELF3* | ERBB2 signaling pathway |
| *ENG* | NfκB |
| *EP300* | Nrf2 |
| *EPAS1* | HIF |
| *EPHA1* | PTK |
| *EPHA2* | PTK |
| *EPHA3* | PTK |
| *EPHA4* | PTK |
| *EPHA5* | PTK |
| *EPHA6* | PTK |
| *EPHA7* | PTK |
| *EPHA8* | PTK |
| *EPHB1* | PTK |
| *EPHB2* | PTK |
| *EPHB3* | PTK |
| *EPHB4* | PTK |
| *EPHB6* | PTK |
| *EPHX1* | Nrf2 |
| *EPM2A* | PTP |
| *ERBB2* | PTK |
| *ERBB3* | PTK |
| *ERBB4* | PTK |
| *ESD* | Nrf2 |
| *ETS1* | TFs |
| *EYA1* | PTP |
| *FASLG* | NfκB |
| *FER* | PTK |
| *FES* | PTK |
| *FGFR1* | PTK |
| *FGFR2* | PTK |
| *FGFR3* | PTK |
| *FGFR4* | PTK |
| *FGR* | PTK |
| *FLT1* | MAPK Signaling |
| *FLT3* | MAPK Signaling |
| *FLT4* | MAPK Signaling |
| *FN1* | NfκB |
| *FOS* | AP_1 |
| *FOSB* | AP_1 |
| *FOSL1* | AP_1 |
| *FOSL2* | AP_1 |
| *FOXO1* | FOXO |
| *FOXO3* | FOXO |
| *FRK* | PTK |
| *FTH1* | Nrf2 |
| *FTL* | Nrf2 |
| *FYN* | PTK |
| *GPX1* | Antioxidant enzymes |
| *GPX2* | Antioxidant enzymes |
| *GPX3* | Antioxidant enzymes |
| *GPX4* | Antioxidant enzymes |
| *GPX5* | Antioxidant enzymes |
| *GPX6* | Antioxidant enzymes |
| *GPX7* | Antioxidant enzymes |
| *GSK3A* | Nrf2 |
| *GSK3B* | Nrf2 |
| *GSR* | Antioxidant enzymes |
| *GSTA1* | Antioxidant enzymes |
| *GSTA2* | Antioxidant enzymes |
| *GSTA3* | Antioxidant enzymes |
| *GSTA4* | Antioxidant enzymes |
| *GSTA5* | Antioxidant enzymes |
| *GSTK1* | Antioxidant enzymes |
| *GSTM1* | Antioxidant enzymes |
| *GSTM2* | Antioxidant enzymes |
| *GSTM3* | Antioxidant enzymes |
| *GSTM4* | Antioxidant enzymes |
| *GSTM5* | Antioxidant enzymes |
| *GSTO1* | Antioxidant enzymes |
| *GSTO2* | Antioxidant enzymes |
| *GSTP1* | Antioxidant enzymes |
| *GSTT1* | Antioxidant enzymes |
| *GSTTP1* | Antioxidant enzymes |
| *GSTZ1* | Antioxidant enzymes |
| *HCK* | PTK |
| *HIF1A* | HIF |
| *HIF3A* | HIF |
| *HMOX1* | Nrf2 |
| *HP* | Nrf2 |
| *HPGDS* | Nrf2 |
| *HRAS* | MAPK |
| *HSP90AA1* | PI3K |
| *HSP90AB1* | PI3K |
| *HSPB1* | FOXO |
| *ICAM1* | NfκB |
| *IER3* | NfκB |
| *IGF1R* | PTK |
| *IKBIP* | NfκB |
| *IKBKB* | NfκB |
| *IKBKE* | NfκB |
| *INPP5D* | PI3K |
| *INSR* | PTK |
| *IRAK2* | NfκB |
| *IRAK3* | NfκB |
| *ITK* | PTK |
| *JAK1* | PTK |
| *JAK2* | PTK |
| *JAK3* | PTK |
| *JDP2* | AP-1 |
| *JUN* | APE/Ref-1 |
| *JUNB* | AP-1 |
| *JUND* | AP-1 |
| *KDR* | MAPK Signaling |
| *KEAP1* | Nrf2 |
| *KIT* | PTK |
| *KRAS* | MAPK |
| *KRT1* | Wnt/Beta-catenin |
| *KSR1* | MAPK |
| *KSR2* | MAPK |
| *LCK* | PTK |
| *LMTK2* | PTK |
| *LMTK3* | PTK |
| *LTK* | PTK |
| *LYN* | PTK |
| *MADCAM1* | NfκB |
| *MAF* | AP_1 |
| *MAFB* | AP_1 |
| *MAFF* | AP_1 |
| *MAFG* | AP_1 |
| *MAFK* | AP_1 |
| *MAP2K1* | MAPK Signaling |
| *MAP2K2* | MAPK Signaling |
| *MAP2K3* | MAPK Signaling |
| *MAP2K4* | MAPK Signaling |
| *MAP2K5* | MAPK Signaling |
| *MAP2K6* | MAPK Signaling |
| *MAP2K7* | MAPK Signaling |
| *MAP3K1* | MAPK Signaling |
| *MAP3K11* | MAPK Signaling |
| *MAP3K14* | MAPK Signaling |
| *MAP3K2* | MAPK Signaling |
| *MAP3K3* | MAPK Signaling |
| *MAP3K5* | MAPK Signaling |
| *MAP3K7* | MAPK Signaling |
| *MAP3K8* | MAPK Signaling |
| *MAPK1* | MAPK Signaling |
| *MAPK10* | MAPK Signaling |
| *MAPK14* | MAPK Signaling |
| *MAPK3* | MAPK Signaling |
| *MAPK4* | MAPK Signaling |
| *MAPK6* | MAPK Signaling |
| *MAPK7* | MAPK Signaling |
| *MAPK8* | MAPK Signaling |
| *MAPK8IP1* | MAPK Signaling |
| *MAPK9* | MAPK Signaling |
| *MATK* | PTK |
| *MDM2* | HIF |
| *ME1* | Nrf2 |
| *MERTK* | PTK |
| *MET* | PTK |
| *MGST1* | Antioxidant enzymes |
| *MGST2* | Antioxidant enzymes |
| *MGST3* | Antioxidant enzymes |
| *MMP3* | NfκB |
| *MMP9* | NfκB |
| *MST1* | FOXO |
| *MST1R* | PTK |
| *MTMR10* | PTP |
| *MTMR11* | PTP |
| *MTMR12* | PTP |
| *MTMR14* | PTP |
| *MTMR15* | PTP |
| *MTMR2* | PTP |
| *MTMR3* | PTP |
| *MTMR4* | PTP |
| *MTMR6* | PTP |
| *MTMR7* | PTP |
| *MTMR9* | PTP |
| *MTOR* | PI3K |
| *MUC1* | MAPK Signaling |
| *MUSK* | PTK |
| *MYB* | TFs |
| *MYC* | NfκB |
| *NFAT5* | TFs |
| *NFATC1* | TFs |
| *NFATC2* | TFs |
| *NFATC3* | TFs |
| *NFATC4* | TFs |
| *NFE2L2* | Nrf2 |
| *NFΚB1* | APE/Ref-1 |
| *NFΚB2* | APE/Ref-1 |
| *NOS1* | ROS Source |
| *NOS2* | ROS Source |
| *NOS3* | ROS Source |
| *NOX3* | ROS Source |
| *NOX4* | ROS Source |
| *NQO1* | Nrf2 |
| *NQO2* | Nrf2 |
| *NR3C1* | TFs |
| *NRAS* | MAPK |
| *NRL* | AP-1 |
| *NTRK1* | PTK |
| *NTRK2* | PTK |
| *NTRK3* | PTK |
| *OGG1* | DNA repair |
| *OS9* | HIF |
| *P4HTM* | HIF |
| *PAK1* | NfκB |
| *PALB2* | Nrf2 |
| *PAX5* | APE/Ref-1 |
| *PAX8* | APE/Ref-1 |
| *PDGFRA* | PTK |
| *PDGFRB* | PTK |
| *PDK1* | PI3K |
| *PEBP1* | APE/Ref-2 |
| *PGD* | Nrf2 |
| *PHLPP1* | PI3K |
| *PHLPP2* | PI3K |
| *PIK3C2A* | PI3K |
| *PIK3C2B* | PI3K |
| *PIK3C2G* | PI3K |
| *PIK3C3* | PI3K |
| *PIK3CA* | PI3K |
| *PIK3CB* | PI3K |
| *PIK3CD* | PI3K |
| *PIK3CG* | PI3K |
| *PIK3R1* | PI3K |
| *PIK3R2* | PI3K |
| *PIK3R3* | PI3K |
| *PIK3R4* | PI3K |
| *PIK3R5* | PI3K |
| *PIK3R6* | PI3K |
| *PKLR* | NfκB |
| *PKN1* | PCK |
| *PKN2* | PCK |
| *PKN3* | PCK |
| *PPARG* | Nuclear Receptors in Lipid Metabolism and Toxicity |
| *PPP2CA* | PI3K |
| *PRDX1* | Antioxidant enzymes |
| *PRDX2* | Antioxidant enzymes |
| *PRDX3* | Antioxidant enzymes |
| *PRDX5* | Antioxidant enzymes |
| *PRDX6* | Antioxidant enzymes |
| *PRKACA* | Transcription Androgen Receptor nuclear signaling |
| *PRKCA* | PKC, MAPK |
| *PRKCB* | PKC, MAPK |
| *PRKCD* | Nrf2 |
| *PRKCE* | PKC, MAPK |
| *PRKCG* | PKC, MAPK |
| *PRKCH* | PKC, MAPK |
| *PRKCI* | PKC, MAPK |
| *PRKCQ* | PKC, MAPK |
| *PRKCZ* | PKC, MAPK |
| *PRKD1* | PKD |
| *PRKD2* | PKD |
| *PRKD3* | PKD |
| *PRKDC* | PI3K |
| *PTEN* | PI3K |
| *PTGS1* | ROS Source |
| *PTGS2* | ROS Source |
| *PTK2* | PTK |
| *PTK2B* | PTK |
| *PTK6* | PTK |
| *PTK7* | PTK |
| *PTP4A1* | PTP |
| *PTP4A2* | PTP |
| *PTP4A3* | PTP |
| *PTPDC1* | PAK pathway |
| *PTPN1* | PTP |
| *PTPN11* | PTP |
| *PTPN12* | PTP |
| *PTPN13* | NfκB |
| *PTPN14* | PTP |
| *PTPN18* | PTP |
| *PTPN2* | PTP |
| *PTPN21* | PTP |
| *PTPN22* | PTP |
| *PTPN23* | PTP |
| *PTPN3* | PTP |
| *PTPN4* | PTP |
| *PTPN5* | PTP |
| *PTPN6* | PTP |
| *PTPN7* | PTP |
| *PTPN9* | PTP |
| *PTPRA* | PTP |
| *PTPRB* | PTP |
| *PTPRC* | PTP |
| *PTPRD* | PTP |
| *PTPRE* | PTP |
| *PTPRF* | PTP |
| *PTPRG* | PTP |
| *PTPRH* | PTP |
| *PTPRJ* | PTP |
| *PTPRK* | PTP |
| *PTPRM* | PTP |
| *PTPRN* | PTP |
| *PTPRN2* | PTP |
| *PTPRO* | PTP |
| *PTPRQ* | PTP |
| *PTPRR* | PTP |
| *PTPRS* | PTP |
| *PTPRT* | PTP |
| *PTPRU* | PTP |
| *PTPRV* | PTP |
| *PTPRZ1* | PAK Pathway |
| *RAF1* | MAPK |
| *RBX1* | Nrf2 |
| *REL* | APE_Ref_1 |
| *RELA* | APE_Ref_1 |
| *RELB* | APE_Ref_1 |
| *RNGTT* | PTP |
| *ROR1* | PTK |
| *ROR2* | PTK |
| *RPS6KB1* | HIF |
| *RSPO1* | Wnt/Beta-catenin |
| *RUNX1* | APE_Ref_1 |
| *RUNX2* | APE_Ref_1 |
| *RUNX3* | APE_Ref_1 |
| *RYK* | PTK |
| *SBF1* | Membrane trafficking |
| *SBF2* | Membrane trafficking |
| *SELE* | NfκB |
| *SFN* | FOXO |
| *SIRT1* | Nrf2 |
| *SKP2* | FOXO |
| *SMAD1* | SMAD |
| *SMAD2* | SMAD |
| *SMAD3* | SMAD |
| *SMAD4* | SMAD |
| *SMAD5* | SMAD |
| *SMAD6* | SMAD |
| *SMAD7* | SMAD |
| *SMAD9* | SMAD |
| *SOD1* | Antioxidant enzymes |
| *SOD2* | Antioxidant enzymes |
| *SOD3* | Antioxidant enzymes |
| *SP1* | TFs |
| *SQSTM1* | Nrf2 |
| *SRC* | PTK |
| *SRMS* | PTK |
| *SRXN1* | Nrf2 |
| *SSH1* | PTP |
| *SSH2* | PTP |
| *SSH3* | PTP |
| *STAT1* | STAT |
| *STAT2* | STAT |
| *STAT3* | STAT |
| *STAT4* | STAT |
| *STAT5A* | STAT |
| *STAT5B* | STAT |
| *STYK1* | PTK |
| *STYX* | PTP |
| *STYXL1* | PTP |
| *SYK* | PTK |
| *TBK1* | NfκB |
| *TEC* | PTK |
| *TEK* | PTK |
| *TENC1* | PTP |
| *THEM4* | PI3K |
| *TIE1* | PTK |
| *TNC* | NfκB |
| *TNFAIP3* | NfκB |
| *TNK1* | PTK |
| *TNK2* | PTK |
| *TNS1* | Integrin pathway |
| *TPTE* | PTP |
| *TPTE2* | PTP |
| *TRAF1* | NfκB |
| *TRAF2* | NfκB |
| *TRAF6* | NfκB |
| *TRIM23* | HIF |
| *TTF1* | APE_Ref_1 |
| *TXK* | PTK |
| *TXN* | Nrf2 |
| *TXNRD1* | Nrf2 |
| *TYK2* | PTK |
| *TYRO3* | PTK |
| *USF1* | TFs |
| *VCAM1* | NfκB |
| *VHL* | Cellular Senescence |
| *WNT1* | Wnt/Beta-catenin |
| *WNT10A* | Wnt/Beta-catenin |
| *WNT10B* | Wnt/Beta-catenin |
| *WNT11* | Wnt/Beta-catenin |
| *WNT16* | Wnt/Beta-catenin  Wnt/Beta-catenin |
| *WNT2* | Wnt/Beta-catenin |
| *WNT2B* | Wnt/Beta-catenin |
| *WNT3* | Wnt/Beta-catenin |
| *WNT3A* | Wnt/Beta-catenin |
| *WNT4* | Wnt/Beta-catenin |
| *WNT5A* | Wnt/Beta-catenin |
| *WNT5B* | Wnt/Beta-catenin |
| *WNT6* | Wnt/Beta-catenin |
| *WNT7A* | Wnt/Beta-catenin |
| *WNT7B* | Wnt/Beta-catenin |
| *WNT8A* | Wnt/Beta-catenin |
| *WNT9A* | Wnt/Beta-catenin |
| *WNT9B* | Wnt/Beta-catenin |
| *XDH* | ROS Source |
| *YAP1* | TFs |
| *YES1* | PTK |
| *YWHAB* | FOXO |
| *YWHAE* | FOXO |
| *YWHAG* | FOXO |
| *YWHAH* | FOXO |
| *YWHAQ* | FOXO |
| *YWHAZ* | FOXO |
| *ZAP70* | PTK |

**Table S2 Top 10 CpG sites with respect to associations with 8-isoprostane concentrations in the derivation set**

| **Identified  CpG sites** | **Gene  name** | **Pathways** | **ß-coefficient** | **SE** | ***P-*value** | **FDR** |
| --- | --- | --- | --- | --- | --- | --- |
|  |  |  |  |  |  |  |
| cg15093079 | *EPHA6* | EPHA forward signaling pathway | 5.16 | 1.36 | 7.56x10^-5^ | 0.180 |
| cg08862778 | *MTOR* | Various pathways, such as PI3K/Akt | -4.36 | 1.31 | 1.01x10^-4^ | 0.180 |
| cg19192120 | *SSH3* | Regulation of actin cytoskeleton and Cytoskeletal signaling pathways | 2.34 | 0.65 | 1.42x10^-4^ | 0.180 |
| cg19623877 | *MYB* | Response to elevated platelet cytosolic Ca^2+^ | 3.56 | 1.44 | 2.09x10^‑4^ | 0.199 |
| cg01009697 | *NTRK2* | PI3K/Akt and MAPK signaling pathways | 4.24 | 1.12 | 3.00x10^-4^ | 0.227 |
| cg06671842 | *PTPN5* | PAK and MAPK signaling pathways | 2.23 | 0.65 | 4.43x10^-4^ | 0.227 |
| cg02168857 | *EPHA7* | EPHA forward signaling pathway | 2.87 | 0.76 | 5.12x10^-4^ | 0.227 |
| cg25365794 | *ALOXE3* | Prostaglandin 2 biosynthesis and metabolism | -4.52 | 1.43 | 5.25x10^-4^ | 0.227 |
| cg27095527 | *PPARG* | Nuclear Receptors in Lipid Metabolism and Toxicity | 4.05 | 1.14 | 6.24x10^-4^ | 0.227 |
| cg05784862 | *KSR1* | RET signaling and MAPK signaling pathways | -2.11 | 0.58 | 7.05x10^-4^ | 0.227 |

Abbreviations: Akt, protein kinase B; ALOXE3, Arachidonate Lipoxygenase 3; EPHA6, EPH Receptor A6; EPHA7, EPH Receptor A7; FDR, false discovery rate; KSR1, Kinase Suppressor Of Ras 1; MAPK, Mitogen-activated protein kinase; MTOR, Mechanistic Target Of Rapamycin Kinase; MYB, MYB Proto-Oncogene, Transcription Factor; NTRK2, Neurotrophic Receptor Tyrosine Kinase 2; PI3K, Phosphoinositide 3-kinase; PPARG, Peroxisome Proliferator Activated Receptor Gamma; PTPN5, Protein Tyrosine Phosphatase, Non-Receptor Type 5; SE, standard error; SSH3, Slingshot Protein Phosphatase 3.


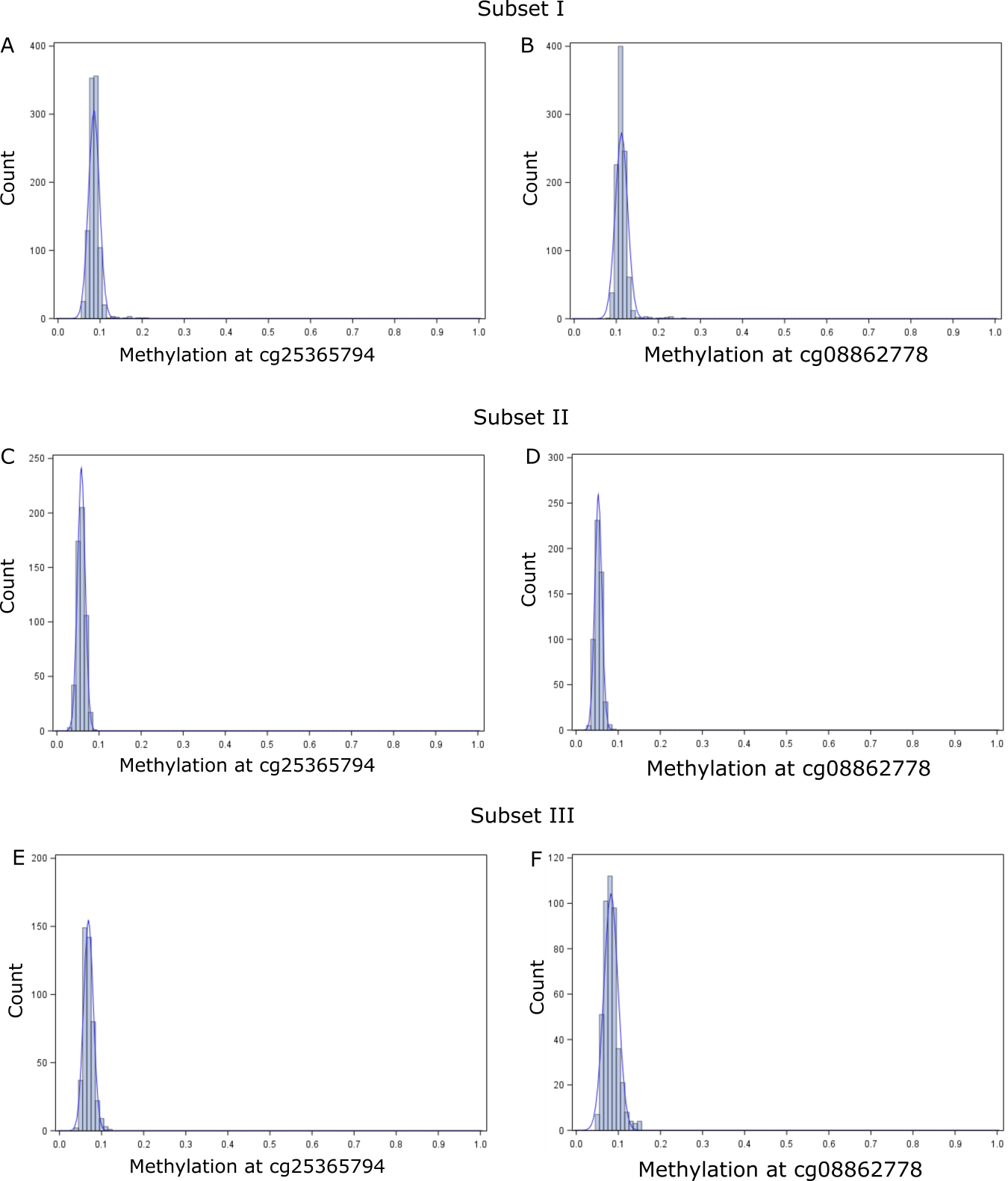


**Figure S1 Distributions of the methylation levels of the selected CpG sites across subsets.** (A) Distribution of DNA methylation at cg25365794 in subset I (deviation set), Median (IQR) = 0.085 (0.079, 0.091); (B) Distribution of DNA methylation at cg08862778 in subset I (deviation set), Median (IQR) = 0.111 (0.105, 0.117); (C) Distribution of DNA methylation at cg25365794 in subset II (1^st^ validation set), Median (IQR) = 0.058 (0.052, 0.064); (D) Distribution of DNA methylation at cg08862778 in subset II (1^st^ validation set), Median (IQR) = 0.053 (0.047, 0.058); (E) Distribution of DNA methylation at cg25365794 in subset III (2^nd^ validation set), Median (IQR) = 0.067 (0.060, 0.075); (F) Distribution of DNA methylation at cg08862778 in subset III (2^nd^ validation set), Median (IQR) = 0.080 (0.071, 0.091).


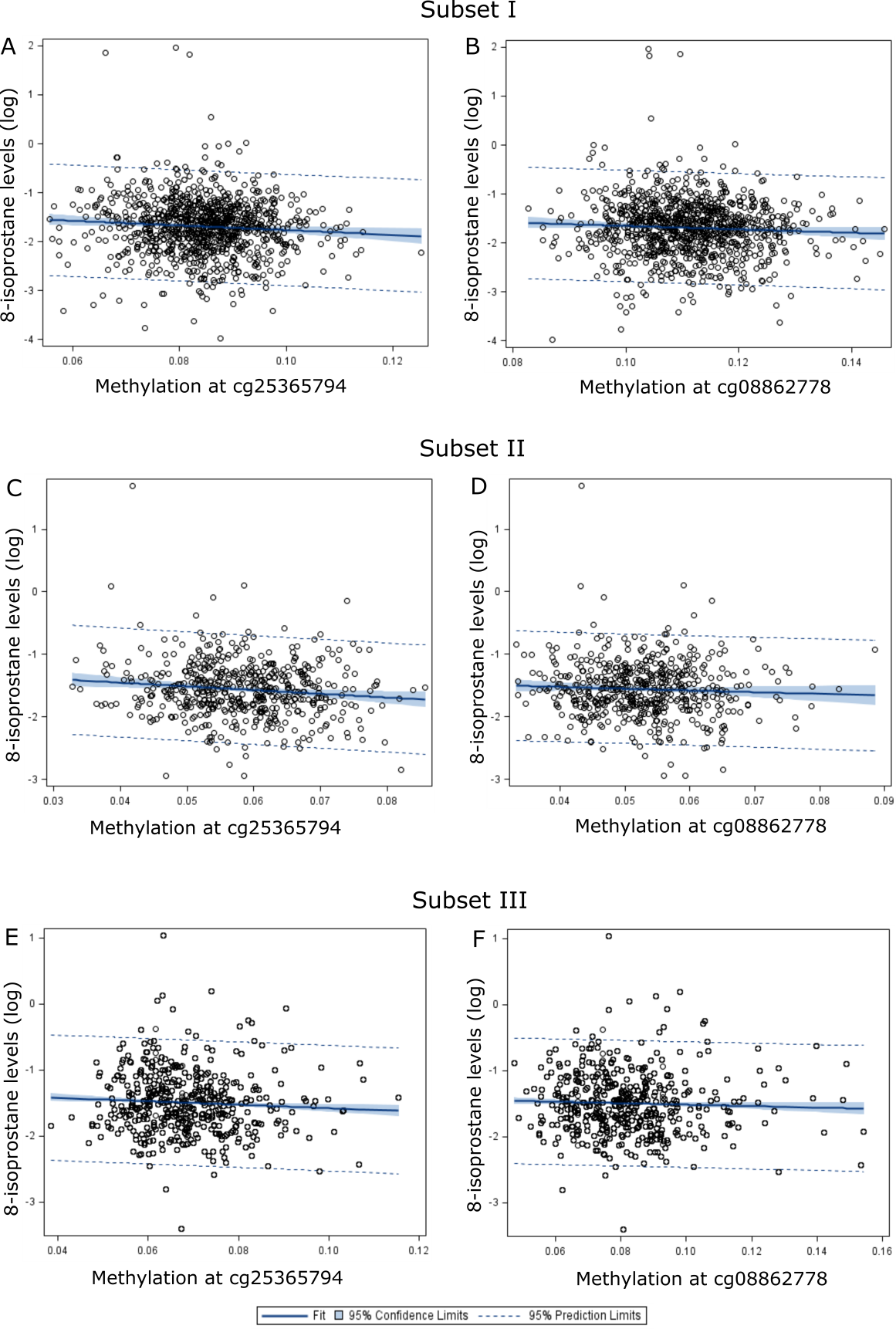


**Figure S2 Scatter plots showing linear associations between DNA methylation at two selected CpG sites and 8-isoprostane levels (Log-transformation) across subsets.** (A) Explained variance by DNA methylation at cg25365794 of 8-isoprostane levels in subset I (deviation set), R^2^ = 0.0062; (B) Explained variance by DNA methylation at cg08862778 of 8-isoprostane levels in subset I (deviation set), R^2^ = 0.0033; (C) Explained variance by DNA methylation at cg25365794 of 8-isoprostane levels in subset II (1st validation set), R^2^ = 0.0148; (D) Explained variance by DNA methylation at cg08862778 of 8-isoprostane levels in subset II (1st validation set), R^2^ = 0.0028; (E) Explained variance by DNA methylation at cg25365794 of 8-isoprostane levels in subset III (2nd validation set), R^2^ = 0.0039; (F) Explained variance by DNA methylation at cg08862778 of 8-isoprostane levels in subset III (2nd validation set), R^2^ = 0.0015.
